# Supplementary material for: Effects of Cinnamomum zeylanicum (Ceylon cinnamon) extract on lipid profile, glucose levels and its safety in adults: A randomized, double-blind, controlled trial
Source: PLoS One. 2025 Jan 24;20(1):e0317904. doi: 10.1371/journal.pone.0317904 (PMC11759401; doi:10.1371/journal.pone.0317904)
Supplement: S2 File — (PDF) [file pone.0317904.s002.pdf]

TITLE: Randomized placebo-controlled trial to determine the effect of a standardized Cinnamon extract on serum cholesterol levels

TITLE: Randomized placebo-controlled trial to determine the effect of a standardized Cinnamon extract on serum cholesterol levels

TRIAL REGISTRATION:

PROTOCOL VERSION: Protocol_V5.1_14 December 2021

FUNDING: SDS Spices (PVT) LTD, 17 Horton Pl, Colombo 00700, Sri Lanka

Table of Contents

LIST OF ABBREVIATIONS ... 3

PROTOCOL SIGNATURE PAGE ... 5

PROTOCOL SYNOPSIS ... 6

1. INTRODUCTION ... 11

1.1 Background ... 11

1.2 Cinnamon ... 12

1.3 Risk/benefit Assessment of the study ... 13

1.4 Rationale ... 15

1.5 Compliance ... 16

2. TRIAL OBJECTIVES ... 16

3. TRIAL POPULATION ... 18

3.1 Inclusion Criteria ... 18

3.2 Exclusion Criteria ... 18

4. STUDY DESIGN ... 20

4.1 Study design and setting ... 20

4.2 Primary and secondary endpoints ... 20

4.3 Outcome measurement ... 21

4.4 Visit details ... 21

4.4.1 Screening ... 21
4.4.2 Randomization ... 22
4.4.3 Follow up visits (Visit 3 and Visit 4) ... 24
4.4.4 Follow up visit (Visit 5 – EOS) ... 24

4.5 Blinding ... 25

4.6 Data collection methods ... 25

4.6.1 Baseline data collection ... 25
4.6.2 Follow up data collection ... 26

4.7 Study duration ... 26

4.8 Discontinuation criteria ... 27

4.9 End of study ... 28

4.10 Termination of the entire trial ... 28

5. INVESTIGATIONAL MEDICINAL PRODUCT ... 29

5.1 Formulation ... 29

5.2 Packaging and Labelling 29
5.3 Storage and Handling 30
5.4 Accountability for Study Drug 30
5.5 Investigational Medicinal Product Return or Disposal 30
6. SAFETY ASSESSMENT 31
6.1 Definitions of Adverse Events, Adverse Reactions, and Serious Adverse Events 31
6.2 Assessment of Adverse Events and Serious Adverse Events 32
6.3 Investigator Requirements and Instructions for Reporting Adverse Events and Serious Adverse Events 33
7. DATA MANAGEMENT 34
8. STATISTICAL CONSIDERATION 34
8.1 Sample size 34
8.2 Statistical Analysis 34
9. INFORMED CONSENT 35
10. RESPONSIBILITIES 35
11. DISSEMINATION OF RESULTS 36
12. CONFIDENTIALITY 36
13. ETHICAL CONSIDERATION 36
14. REFERENCES 37

LIST OF ABBREVIATIONS

ACR	Albumin Creatinine Ratio
AE	Adverse Events
ALD	Alcoholic Liver Disease
ALT	Alanine Aminotransferase
AST	Aspartate Aminotransferase
ASCVD	Atherosclerotic Cardiovascular Disease
AR	Adverse Reaction
BP	Blood pressure
CAD	Coronary Artery Disease
CVD	Cardiovascular Disease
DPB	Diastolic Blood Pressure
eGFR	Estimate Glomerular Filtration rate
FBS	Fasting Blood Sugar
GCP	Good Clinical Practice
HC	Hip circumference
HDL	High-Density Lipoprotein
HDL-C	High-Density Lipoprotein Cholesterol
LDL	Low-Density Lipoprotein
LDL-C	Low-Density Lipoprotein Cholesterol
MI	Myocardial Infarction
NAFLD	Non-alcoholic Fatty Liver Disease

NCD	Non-Communicable Diseases
NCEP-ATP III	National Cholesterol Education Program Expert Panel and Adult Treatment Panel III
SAE	Serious Adverse Event
SBP	Systolic Blood Pressure
sd-LDL	Small and Dense Low-Density Lipoprotein
T2DM	Type 2 Diabetes Mellitus
TC	Total Cholesterol
WC	Waist Circumference
WHR	Waist-hip ratio
WHO	World Health Organization

PROTOCOL SIGNATURE PAGE

Protocol Title: Randomized placebo-controlled trial to determine the effect of a standardized Cinnamon extract on serum cholesterol levels

Protocol No: V5.1_ 14 December 2021

Authorised Sponsor Representative Signature:

Date: __________________ Signature: __________________

I agree with the content of this protocol and the confidential nature of the documentation made as part of this study. I also acknowledge that the Sponsor of the study has the right to discontinue the study at any time. I have read the protocol and understand it and will work according to it, as well as according to the principles of Good Clinical Practice, applicable laws and regulations and the Declaration of Helsinki.

Principal Investigator

Dr Dimuthu Muthukuda
Consultant Endocrinologist
Sri Jayewardenepura General Hospital
Sri Lanka Date: _____________ Signature: _____________________

Principal Trial Statistician /Investigator
Prof. A. Pathmeswaran
Senior Professor in Public Health
Department of Public Health
Faculty of Medicine
University of Kelaniya
Sri Lanka Date: _____________ Signature: _____________________

Study Site Investigator

Study Site Name: ____

Name / Title: ____

Signature: ______________________________

Date: ________________________________

PROTOCOL SYNOPSIS

**Name of Sponsor/Company:**<br>SDS Spices (Pvt) Ltd, 17 Horton Pl, Colombo 00700, Sri Lanka
**Name of Investigational Product:**<br>SaaraLife OptiHeart - Ceylon Cinnamon extract capsule
**Name of Active Ingredient:**<br>Ceylon Cinnamon (*Cinnamomum zeylanicum)* bark extract
**Title of Study:**<br>Randomized placebo-controlled trial to determine the effect of a standardized Cinnamon extract on serum cholesterol levels.
**Study Design:**<br><br>The study will be a randomized, double-blinded, placebo-controlled clinical trial. It will be conducted at several hospitals in Sri Lanka for a period of 3 months, assessing the effects of the daily supplementation of capsules containing *C. zeylanicum* in individuals with average serum LDL level between 100-190 mg/dL (100 mg/dL≤LDL<190 mg/dL). The duration of the trial will be 3 months, based on the evidence from previous research conducted on *C. zeylanicum*, and this duration is expected to be sufficient to identify changes in the primary outcome (LDL level) assessed during the clinical trial.<br><br>*<u>Screening visit (Visit1):</u>*<br><br>Adult volunteers aged between 18 - 70 years who are not on lipid lowering drugs will be screened for eligibility criteria after obtaining their consent. Those who consent will be screened for eligibility into the study. Individuals with average serum LDL level 100-190 mg/dL (100 mg/dL≤LDL<190 mg/dL) will be recruited to the study and a unique study identifier will be given, and baseline data collection will be administered.<br><br>Steps to be followed<br>• Assess the potential participant's interest and eligibility for the trial.<br>• Discuss the trial with the participant, including using the participant information sheet, and obtain signed informed consent. This can also be done before the screening visit.<br>• Collect demographic information.<br>• Collect information on medical history<br>• Measure height, weight, WC, HC, mid arm circumference, SBP, DBP, and pulse.<br>• Assess eligibility as per the trial inclusion and exclusion criteria.<br>• Arrange baseline laboratory investigations<br>– Lipid profile (total cholesterol, HDL, LDL and triglycerides). Investigations done up to 7 days prior to screening visit will be considered to determine the eligibility at screening and as baseline lipid parameters.<br>– FBS and HbA1c<br>– Liver profile (ALT, AST, AP)

- Renal profile (Serum creatinine, ACR, Electrolytes -Na, K, Cl, eGFR) will be performed for all the participants.
- Pregnancy test and contraception review if applicable

If the patient fulfils the eligibility criteria he or she can be randomized on the same day or can randomize within one week.

Randomization – Visit 2

Steps to be followed at Randomization (Visit 2)

- Record any AEs and SAEs that have occurred since the screening visit (if randomization is done in a separate date).
- Measure SBP, DBP and pulse
- Anthropometric measurements – Weight / WC / HC / mid arm circumference.
- Review and record all medication currently being taken by the participant.
- Pregnancy test and contraception review if applicable
- Confirm that participant is suitable to be randomized.
- Randomize participant.
- Prescribe treatment according to group allocation.
- Two weeks of post randomization a compulsory follow up call will be given to all the participants.

Follow up visits (Visit 3 and Visit 4)

Following steps will be carried out during the Visit 3 (1 month) and Visit 4 (2 month)

- Record AEs and SAEs since the previous visit.
- Measure SBP, DBP and pulse
- Anthropometric measurements – Weight / WC / HC / mid arm circumference
- Review and record all medications being taken by the participant.
- Pregnancy test and contraception review if applicable
- Collect unused trial medication and review medication adherence.
- Issue new pack of treatment drug/placebo as per the initial allocation

Follow up visit (Visit 5 – EOS)

At the end of the study

- Record AEs and SAEs since the previous visit and other clinical history.
- Measure SBP, DBP and pulse
- Anthropometric measurements – Weight / WC / HC / mid arm circumference
- Review and record all medications being taken by the participant.
- Collect unused trial medication and review medication adherence.
- Collect blood for investigations

• Record laboratory results (FBS, HbA1c, lipid profile, liver profile, serum creatinine, ACR, Electrolytes-Na, K, Cl, eGFR). Each test should be conducted by the same laboratory that conducted the investigations at the screening and/or baseline.<br>• Collect unused trial medication and review medication adherence.<br>• Any ongoing AE reported at the EOS will be followed up by the investigators until it is resolved.
**Objectives:**<br><br>***Primary***<br>• To evaluate the effect of a standardized *C. zeylanicum* extract on serum LDL levels.<br><br>***Secondary***<br>• To evaluate the effect on other lipid profile parameters (HDL, total cholesterol and triglycerides)<br>• To evaluate the effect on sugar levels (FBS, HbA1c)<br>• To evaluate the effects on anthropometric parameters (weight, WC, HC, mid arm circumference)<br>• To evaluate the effect on systolic blood pressure (SBP), diastolic blood pressures (DBP).<br>• To assess the potential effects of regular administration of *C. zeylanicum* extracts on liver and kidney function and the occurrence of self-reported side effects in individuals under study.
**Number of participants (planned):**<br>Total -150 (75 participants per each active and placebo arm)
**Diagnosis & Main Criteria for Inclusion:**<br>**Inclusion Criteria:**<br><br>• Age between 18-70 years<br>• LDL level; between 100 and 190 mg/dL (100 mg/dL $\leq$ LDL < 190 mg/dL)<br><br>**Key Exclusion Criteria:**<br><br>• Participants with an allergy to cinnamon<br>• Already on cinnamon or any other nutritional / herbal / ayurvedic supplements.<br>• Lactation, pregnancy or unwillingness to use an effective form of birth control for women of childbearing years<br>• Any form of malignancy at present or in the past<br>• Blood dyscrasias<br>• Estimated Glomerular Filtration Rate (eGFR) <60ml/min

- Patients with diagnosed alcoholic liver disease (ALD), decompensated cirrhosis or abnormal baseline liver function tests (ALT, AST - Above three times the upper limit of normal)
- Patients with cardiac, liver, renal or respiratory failure (Annexure 01)
- Patients with ASCVD or any other major critical illnesses.
- Any condition that, in the opinion of the primary investigator, would contraindicate the patient’s participation
- Currently on statins or any other lipid lowering drug or if investigator decides that the participant is in need of an immediate lipid lowering therapy (statin) based on clinical judgement.
- Participant with triglycerides level $\geq$300 mg/dL
- Participants with a history of epilepsy and/or on anti-epileptic drugs

Test Product, Dose and Mode of Administration:
The test product is a capsule containing a standardized Cinnamon bark extract. Each participant included in the treatment arm will be advised to take 2 capsules per day (1000 mg/day); one capsule (500 mg) before breakfast and one capsule before dinner. The investigator may prescribe the same dose to be taken after meals (after breakfast and after dinner) if a participant shows any sign of intolerance due taking the capsule before meals. The capsule will be administered orally.

Duration of Treatment:
3 months

Criteria for Evaluation:
Primary Endpoint

- Reduction in LDL levels after three months of treatment.

Secondary Endpoints

- Reduction in total cholesterol and triglycerides and/or effect on HDL levels
- Effect on serum glucose level related parameters (FBS, Hba1c)
- Effect on anthropometric parameters (weight, WC, HC, mid arm circumference)
- Effect on systolic and diastolic blood pressure.
- Occurrence of adverse events due to regular administration of *C. zeylanicum* extracts on liver and kidney function and the occurrence of self-reported side effects in individuals under study.

Potential Risks to individuals
• To be monitored

Statistical Methods and Data Analysis:
Reduction in LDL level at three months (from the baseline value) will be compared between the two groups – cinnamon vs placebo – using ANCOVA. Intention to treat analysis will be adhered to in analysing the outcome data. Data analysis will be done using version 4.0 of the R statistical software.

1. INTRODUCTION

1.1 Background

Hypercholesterolemia is an important risk factor for atherosclerotic cardiovascular disease (ASCVD), including cerebrovascular disease, coronary heart disease, and peripheral arterial disease. For patients with known ASCVD, cholesterol-lowering leads to a consistent reduction in cardiovascular mortality and cardiovascular events in men and women and middle-aged and older patients. Among patients without cardiovascular disease, the data on reduction in atherosclerotic cardiovascular disease events with statin drugs is also well documented. Patients with triglyceride levels of more than 1000 mg/dl are at increased risk of acute pancreatitis [1].

Hypercholesterolemia is diagnosed by a lipid profile, consisting of measurements of total cholesterol, Low-Density Lipoprotein Cholesterol (LDL-C) (estimated or direct), High-Density Lipoprotein Cholesterol (HDL-C), and triglycerides. This condition is treated with lifestyle modifications such as dietary changes, exercise, and smoking cessation, as well as pharmacologic intervention with statin therapy, fibrates, bile acid sequestrants, nicotinic acid, and selective inhibitors of cholesterol absorption [2]. Statin therapy is effective for both primary and secondary prevention for Coronary artery disease (CAD) and improves a number of perioperative outcomes. Significant reductions in cardiovascular morbidity and mortality from lipid-lowering as well as pleiotropic effects have been demonstrated. However, statins are not adequate therapy for patients with elevated lipoprotein and for patients with very high levels of LDL-C, such as those with familial hyperlipidemia [3]. Further, some patients are intolerant of statins and might benefit from alternatives and/or combination therapies and even lower LCL-C levels. [4].

Hypercholesterolemia is a strong determinant of mortality and morbidity associated with CAD and a major contributor to the global disease burden [5]. Hypercholesterolemia (TC > 200 mg/dL) has been reported to have a prevalence of up to 35% in men and 36% in women from South Asian countries [6]. The prevalence of hypercholesterolemia was 53.6% in Sri Lankan population according to research data in 2010 among nationally representative sample of 4451 subjects over 18 years by a multistage random cluster sampling technique [7]. LDL-C is a well-established marker for the occurrence, recurrence, and severity of CAD. It is the co-primary target for lipid-

lowering therapy as per the National Lipid Association recommendations for cholesterol management. Hypercholesterolemia corresponds with an elevated plasma LDL-C level because LDL is the main carrier of cholesterol in plasma [6].

1.2 Cinnamon

Cinnamon has two main varieties, *Cinnamomum cassia* (also known as *Cinnamomum aromaticum*) *and Cinnamomum zeylanicum* (also known as *Cinnamomum verum*). *C. zeylanicum* is also known as “true cinnamon” or “Ceylon cinnamon”. *C. zeylanicum* is a small tropical tree that is indigenous to Sri Lanka and Southern parts of India [8]. Sri Lanka produces the largest quantity and best quality of cinnamon. One important difference between true cinnamon and cassia cinnamon is their coumarin content [9]. Coumarins can have potentially toxic effects on the liver due to strong anticoagulant properties [10]. The coumarin content in Ceylon cinnamon is negligible, whereas the coumarin level in cassia cinnamon is much higher and can cause health risks if consumed in larger quantities on a regular basis [9]. In addition to its culinary uses, in native Ayurvedic medicine Cinnamon is considered a remedy for respiratory, digestive and gynaecological ailments. Almost every part of the cinnamon tree including the bark, leaves, flowers, fruits and roots, has some medicinal or culinary use. The volatile oils obtained from the bark, leaf, and root barks vary significantly in chemical composition, which suggests that they might vary in their pharmacological effects as well [11]. It is also generally recognized as safe when used in therapeutic doses. According to the United States Food and Drug Administration (FDA), the amount of cinnamon in commonly found foods are generally safe and well tolerated [12]. Several preclinical *in vivo* studies also have not shown any significant toxic effects of cinnamon [8].

Different parts of cinnamon such as leaves, bark, root bark and fruits have various amount of resinous compounds [13]. Cinnamaldehyde, cinnamate and cinnamic acid are the main resinous ingredients found in cinnamon that increase in quantity when cinnamon ages. Cinnamaldehyde is responsible for its spicy taste and fragrance. Essential oils, such as trans-cinnamaldehyde, cinnamyl acetate and eugenol are found in cinnamon [13].

Table 2: Chemical contents of different parts of cinnamon [14]

Parts of cinnamon	Dominant ingredient (s)
Leaves	Eugenol: 70.00 to 95.00%
Bark	Cinnamaldehyde: 65.00 to 80.00%
Root bark	Camphor: 60.00%
Fruit	*trans*-cinnamyl acetate 42.00 to 54.00%
Buds	Terpene hydrocarbons: 78.00%<br><br>*alpha*-Bergamotene: 27.38%
Flowers	(E)-cinnamyl acetate: 41.98%<br><br>*trans*-*alpha*-bergamotene: 7.97%

Based on traditional medicine and recent scientific evidence cinnamon and its active ingredients such as cinnamaldehyde, cinnamate, cinnamic acid and eugenol in the forms of aqueous and alcoholic extracts have a variety of therapeutic effects. Certain *in vitro* and *in vivo* trials have mainly shown antioxidant, anti-inflammatory, anti-diabetic, anti-microbial, anti-cancer activities and cholesterol and lipid lowering effect [14].

1.3 Risk/benefit Assessment of the study

South Asians including Sri Lankans have a high prevalence of cardiovascular diseases (CVD) and suffer from early-onset CVD compared to other ethnic groups. South Asians have a unique lipid profile which may predispose them to premature CVD [15]. The hypercholesterolemia in South Asians is characterized by elevated levels of triglycerides, low levels of high-density lipoprotein (HDL) cholesterol, elevated lipoprotein(a) levels, and a higher atherogenic particle burden despite comparable low-density lipoprotein cholesterol levels compared with other ethnic subgroups. HDL particles also appear to be smaller, dysfunctional, and proatherogenic in South Asians [15]. Given the consistent findings of increased prevalence, premature onset, and increased mortality from CVD in South Asians, there has been much interest in determining the underlying causes. Conventional risk factors such as hypertension, hypercholesterolemia, diabetes mellitus,

abdominal obesity, metabolic syndrome, and tobacco use have been clearly associated with CVD risk among South Asian populations [16]. Some studies have shown that three in four Sri Lankan adults have some form of dyslipidemia [17]. Recent data from WHO estimated that cardiovascular diseases account for 40% of deaths in Sri Lanka [18]. It is estimated that 25.4% of Sri Lankan adults are at high risk for cardiovascular disease [19]. Dyslipidemia is a key modifiable risk factor for cardiovascular disease and data on its prevalence in the country and region were scarce [17].

Among most of the plant based traditional medicines 'true cinnamon' plays a key role as a hypolipidemic agent. Several pre-clinical and clinical trials have conducted on this and have shown potential beneficial effects of 'true cinnamon'. A study conducted by Hasan et al. [20], has demonstrated that *C. zeylanicum* reduced total cholesterol, LDL cholesterol and triglycerides while increasing HDL-cholesterol in diabetic rats. Similar results have also been observed in hyper-lipidaemic albino rabbits [21]. A study conducted using hyperlipidaemic albino rabbits showed that *C. zeylanicum* bark powder methanol extract equivalent to 0.75g/kg bark powder and simvastatin (0.6 mg/kg b. wt.) were equieffective in treating hyperlipidaemia [22]. Also, another study conducted using hyperlipidemic albino rats showed a significant ($p<0.05$) hypolipidemic effect on cholesterol and triglycerides of cinnamon extract in groups administered 250 and 500 mg/kg compared with the negative and positive control group. Additionally, the hepatic histopathological study showed that the cinnamon ethanol extract has preventive effect from liver fatty infiltration and granular degeneration [23].

A phase I clinical trial conducted by Ranasinghe et al., [24] has demonstrated that there is a beneficial anti-hyperlipidaemic and blood pressure lowering effects among healthy adults. It had also shown that there are no significant toxic effect or serious adverse effects of true cinnamon (*C. zeylanicum*) other than dyspeptic symptoms Also, that capsules with 500mg of Cinnamon is safe for ingestion and no side effects were reported in the participant cohort. Jain G S et al. [25] reported that a single supplement intervention with 3 g cinnamon for 16 weeks resulted in significant improvements in all components of metabolic syndrome in a sample of Asian Indians in north India. Khan A et al. [26] investigated whether cinnamon improves blood glucose, triglyceride, total cholesterol, HDL cholesterol, and LDL cholesterol levels in people with type 2 diabetes in Pakistan. They found that after 40 days, all three levels of cinnamon (1, 3, and 6 g) reduced the

mean fasting blood sugar (FBS) (18 –29%), triglyceride (23–30%), LDL cholesterol (7–27%), and total cholesterol (12– 26%) levels.

C. zeylanicum has also been shown to have hepato-protective effects in a study where liver injury was induced in rats by CCl4 [27]. Administration of *C. zeylanicum* extracts (0.01, 0.05 and 0.1 g/kg) for 28 days significantly reduced the impact of CCl4 toxicity on the serum markers of liver damage (AST, ALT and ALP). Also, Askari F et al [28] conducted a double-blind, placebo-controlled trial in Iran with two parallel groups to receive daily supplementation with either two capsules of cinnamon (each capsule contain 750 mg cinnamon) or 2 placebo capsules, daily for 12 weeks. Study has shown that 1.5 g of cinnamon supplementation for 12 weeks improved Nonalcoholic fatty liver disease (NAFLD) characteristics and could be a good adjuvant therapeutic option for NAFLD.

Thus, true cinnamon bark extract can be considered as a therapeutic agent for hypercholesterolemia with comparatively less side effects.

1.4 Rationale

Although certain *in vitro* and *in vivo* studies and very few clinical trials have suggested *C. zeylanicum* seems to lower serum lipid levels, suggesting beneficial cardiovascular effects, it was noted the absence of properly conducted randomized controlled human trials, to decide the lipid lowering effect of *C. zeylanicum* in humans and to determine whether these effects have any other health implications. Thus, this double blinded, placebo-controlled study will be conducted to evaluate the potential effects of *C. zeylanicum* extract in individuals with hypercholesterolemia in Sri Lanka whose LDL level is between 100 mg/dL-190 mg/dL (100 mg/dL < LDL < 190 mg/dL). As per AHA (2018) guidelines, anyone with LDL level >190mg/dL should be started with statins. For any individual with LDL level ≥70mg/dL <190mg/dL and without diabetes mellitus, statins will be prescribed only after doing a risk assessment. Thus, the upper cut off will be considered 190 mg/dL. The optimum LDL level is considered as <100 mg/dL and for high-risk individuals (individuals with diabetes or MI) is considered as <70mg/dL. Since people in high-risk category are excluded, the lower cut off was considered as 100mg/dL.

As per AHA (2018) guidelines, since LDL level is considered as the primary parameter in prescribing statins, only LDL level will be considered as an inclusion criterion. Total cholesterol

and triglycerides levels will be not considered as inclusion criteria in recruiting participants into the study.

For triglycerides, the level ≥500 mg/dL is considered as very high level that can cause acute pancreatitis. However, considering the risk factors that can be associated with high level of triglycerides, anyone with triglyceride level ≥300mg/dL will be excluded from the study. This step will be taken to prevent recruiting any participant with a triglycerides level <500 mg/dL but need to be on a lipid lowering drug based on the clinical assessment.

Further to above criteria, if the investigator decides that a particular person is in need of an immediate lipid lowering therapy (statin) based on his/her clinical judgement, such participants will also be excluded and he or she will be directed to proper medication regime. Thus, high risk participants who are in need of immediate statin therapy will not get deprived of those drugs.

The use of placebo in this type of design has an important value, as "placebo response" is widely known to occur during clinical studies. The addition of a placebo arm is important, as this enables a control for potential influences derived from overall medical care. Therefore, the inclusion of a placebo arm in this study is necessary to prove efficacy of Cinnamon capsule.

1.5 Compliance

This study will be conducted in compliance with this protocol, Good Clinical Practice (GCP), and all applicable regulatory requirements.

2. TRIAL OBJECTIVES

The study will evaluate the potential effects of a standardized *Cinnamomum zeylanicum* extract on serum cholesterol levels

Primary Objective

Primary objective of this study is

- To evaluate the effect of a standardized *C. zeylanicum* extract on LDL levels in individuals with average LDL level between 100-190 mg/dL (100 mg/dL ≤ LDL < 190 mg/dL)

Secondary Objectives

Secondary objectives of the study are

- To evaluate the effect on other lipid profile parameters (HDL, total cholesterol and triglycerides)
- To evaluate the effect on sugar levels (FBS, HbA1c)
- To evaluate the effects on anthropometric parameters (weight, waist circumference (WC), hip circumference (HC), mid arm circumference)
- To evaluate the effect on systolic blood pressure (SBP), diastolic blood pressures (DBP).
- To assess the potential effects of regular administration of *C. zeylanicum* extracts on liver and kidney function and the occurrence of self-reported side effects in individuals under study.

3. TRIAL POPULATION

Participants will be recruited on a voluntary basis from a cohort of individuals with average LDL level between 100-190 mg/dL (100 mg/dL $\leq$ LDL < 190 mg/dL). The study will be conducted at several hospitals in Sri Lanka. Informed written consent will be obtained from all study participants. Details of the inclusion and exclusion criteria are given below.

3.1 Inclusion Criteria

Subjects will be eligible if they meet all the following inclusion criteria:

- Age between 18-70 years
- LDL level; between 100 and 190 mg/dL (100 mg/dL $\leq$ LDL < 190mg/dL)

3.2 Exclusion Criteria

Subjects who meet any of the following exclusion criteria are not to be enrolled in the study.

- Participants with an allergy to cinnamon
- Already on cinnamon or any other nutritional / herbal / ayurvedic supplements.
- Lactation, pregnancy or unwillingness to use an effective form of birth control for women of childbearing years
- Any form of malignancy at present or in the past
- Blood dyscrasias
- Estimated Glomerular Filtration Rate (eGFR) <60ml/min
- Patients with diagnosed alcoholic liver disease (ALD), decompensated cirrhosis or abnormal baseline liver function tests (ALT, AST - Above three times the upper limit of normal)
- Patients with cardiac, liver, renal or respiratory failure (Annexure 01)
- Patients with ASCVD or any other major critical illnesses.
- Any condition that, in the opinion of the primary investigator, would contraindicate the patient’s participation

- Currently on statins or any other lipid lowering drug or if investigator decides that the participant is in need of an immediate lipid lowering therapy (statin) based on clinical judgement.
- Participant with triglycerides level $\geq$300 mg/dL
- Participants with a history of epilepsy and/or on anti-epileptic drugs

4. STUDY DESIGN

4.1 Study design and setting

The study will be a randomized, double-blinded, placebo-controlled clinical trial. It will be conducted at several hospitals in Sri Lanka for a period of 3 months, assessing the effects of the daily supplementation of capsules containing *C. zeylanicum* in individuals with average LDL level between 100-190 mg/dL (100 mg/dL $\leq$ LDL < 190 mg/dL).. The duration of the trial will be 3 months, based on the evidence from previous research conducted on *C. zeylanicum*, and this duration is expected to be sufficient to identify changes in the primary outcome (LDL level) assessed during the clinical trial.

4.2 Primary and secondary endpoints

Primary endpoint

The primary endpoint is:

- Reduction in LDL levels after three months of treatment.

Secondary endpoint

- Reduction in total cholesterol and triglycerides and/or effect on HDL levels
- Effect on serum glucose level related parameters (FBS, HbA1c)
- Effect on anthropometric parameters (weight, WC, HC, mid arm circumference)
- Effect on systolic and diastolic blood pressure.
- Occurrence of adverse events due to regular administration of *C. zeylanicum* extracts on liver and kidney function and the occurrence of self-reported side effects in individuals under study.

4.3 Outcome measurement

Outcomes of the study will be assessed at the end of the 3 months of the study. Anthropometric measures, SBP, DBP, FBS, serum lipids, serum creatinine, eGFR, albumin creatinine ratio (ACR) and liver profile (ALT, AST, AP) will be assessed at Visit 05. Drug compliance and any reasons for withdrawal from the study will also be recorded.

Primary effectiveness measures

- Change in LDL levels from baseline to final follow-up at three months post randomization.

Secondary effectiveness measures

- Changes in other lipid profile parameters (HDL, total cholesterol and Triglycerides)
- Changes in FBS and HbA1c
- Changes in anthropometric parameters
- Change in SBP and DBP.
- Potential effects of regular administration of *C. zeylanicum* extracts on liver and kidney function and the occurrence of self-reported side effects in participants of the study.

4.4 Visit details

4.4.1 Screening

Adult volunteers aged between 18 - 70 years who are not on lipid lowering drugs will be screened for eligibility criteria after obtaining their consent. Informed consent will be obtained from all the participants for participation in the study. The participants will be required to sign or print thumb impression (those unable to sign). Those who consent will be screened for eligibility into the study with Screening Form. Individuals with average serum LDL level 100-190mg/dL will be recruited to the study considering other exclusion criteria and an unique study identifier will be given, and baseline data collection will be administered. All women of child-bearing potential must have a

negative highly sensitive pregnancy test 7 days or fewer before starting medication and must use a highly effective method of contraception throughout their participation in the study. Laboratory investigations will be conducted for the participants who are willing to participate in the study. Lipid profiles done up to 7 days prior to screening visit will be considered to determine the eligibility at screening and as baseline lipid parameters.

Steps to be followed at screening (Visit 1)

- Assess the potential participant's interest and eligibility for the trial.
- Discuss the trial with the participant, including using the participant information sheet, and obtain signed informed consent. This can also be done before the screening visit.
- Perform pregnancy test & contraception review if applicable
- Collect demographic information.
- Collect information on medical history
- Measure height, weight, WC, HC, mid arm circumference, SBP, DBP, and pulse (two BP readings will be taken with minimum of 3 min gap).
- Assess eligibility as per the trial inclusion and exclusion criteria.
- Arrange laboratory investigations, including pregnancy, if applicable. The laboratory investigations
  - Lipid profile (total cholesterol, HDL, LDL and triglycerides)
  - FBS and HbA1c
  - Liver profile (ALT, AST, AP)
  - Renal profile (Serum creatinine, ACR, Electrolytes-Na, K, Cl, eGFR) will be performed for all the participants.

If the patient fulfils the eligibility criteria he or she can be randomized on the same day or can randomize within one week.

4.4.2 Randomization

The subjects will be allocated to either cinnamon intervention or placebo group with an allocation ratio of 1:1 using random allocation sequence generated by the R software 'blockrand' package

[30]. Allocation concealment will be done by using pre-packed sequentially numbered containers for each patient. All the containers having the study drug, or the placebo will be identical in appearance with similar looking capsules, equal in weight and tamper– proof. Six bottles (each bottle with 35 capsules) with same code will be prepared for each patient for the treatment period and will be given sequentially. The investigators and participants will remain blinded to treatment allocation until the outcomes is analysed. The participants will be enrolled, assigned to the intervention and followed up throughout the study by the clinical team.

Post randomization, the participants will be assigned to either the cinnamon intervention group (1000 mg/daily) or the placebo group (Wheat flour -white colour tasteless odourless fine powder-1000mg/daily) receiving the intervention in the form of capsules for 12 weeks. Each subject in both groups will be instructed to consume one capsules (500 mg) before breakfast and one capsule (500 mg) before dinner as well as continue with the diet and physical activity recommended to them by the investigator. The investigator may prescribe the same dose to be taken after meals (after breakfast and after dinner) if a participant shows any sign of intolerance due to taking the capsule before meals.

Weight, WC, HC, mid arm circumference, HbA1c, liver profile (AST, ALT and AP), serum creatinine level, ACR, electrolytes-Na, K, Cl, eGFR, SBP, DBP and pulse will be assessed before and after the intervention. Two cinnamon or placebo capsule bottles will be given every 4 weeks to the participants of the respective groups on their monthly visit to the clinic. Compliance to the protocol will be monitored in each visit. Participants will be advised to bring back the empty bottles with the left-over capsules (if any) at the time of the monthly visit to the clinic.

Steps to be followed at Randomization (Visit 2)

- Record any AEs that have occurred since the screening visit (if randomization is done in a separate date).
- Perform pregnancy test & contraception review if applicable. If the participant is positive, then terminate the participant without randomization.
- Measure SBP, DBP and pulse (two BP readings will be taken with minimum of 3 min gap)
- Anthropometric measurements – Weight / WC / HC / mid arm circumference
- Review and record all medications currently being taken by the participant.

- Laboratory results obtained at screening will be considered as Baseline results – Lipid profiles done up to 7 days prior to screening visit will be considered to determine the eligibility at screening and as baseline lipid parameters.
-
- Confirm that participant is suitable to be randomized.
- Randomize participant.
- Prescribe treatment according to group allocation.
- Two weeks of post randomization a compulsory follow up call will be given to all the participants.

4.4.3 Follow up visits (Visit 3 and Visit 4)

Following steps will be carried out during the Visit 3 (1 month) and Visit 4 (2 month)

- Record AEs and SAEs since the previous visit.
- Perform pregnancy test & contraception review if applicable. If the pregnancy test is positive, then fill the EOS form and terminate the participant.
- Measure SBP, DBP and pulse (two BP readings will be taken with minimum of 3 min gap)
- Anthropometric measurements – Weight / WC / HC / mid arm circumference
- Review and record all medications being taken by the participant.
- Collect unused trial medication and review medication adherence.
- Issue new pack of treatment drug/placebo as per the initial allocation

4.4.4 Follow up visit (Visit 5 – EOS)

At the end of the study

- Record AEs and SAEs since the previous visit.
- Measure SBP, DBP and pulse (two BP readings will be taken with minimum of 3 min gap)
- Anthropometric measurements – Weight / WC / HC / mid arm circumference
- Review and record all medications being taken by the participant.
- Collect unused trial medication and review medication adherence.
- Collect blood for investigations

- Record laboratory results (FBS, HbA1c, lipid profile, liver profile, serum creatinine, ACR, electrolytes, eGFR). Each test should be conducted by the same laboratory that conducted the investigations at the screening and/or baseline.
- Collect unused trial medication and review medication adherence.
- Any ongoing AE reported at the EOS will be followed up by the investigators until it is resolved.

4.5 Blinding

The investigators and participants will be remained blinded to the treatment allocations. The medication will be delivered in similar bottles and labels, each with its own sequence number. Bottles containing a monthly supply of either placebo or cinnamon capsule will be prepared according to the randomization sequence and supplied to the participants by the investigator or research assistants when they are randomized to the trial.

4.6 Data collection methods

4.6.1 Baseline data collection

Those who consent and are eligible will constitute the study sample (n=150 or 75 per arm). Domains of the baseline data collection form include socio-demographic information, vitals (SBP, DBP and pulse), anthropometric measures (Height, Weight, WC, HC and mid arm circumference) will be recorded. Fasting (12 hrs) blood samples (10 ml) will be collected to analyse total cholesterols, HDL, LDL, triglycerides, FBS, HbA1c, liver profile (ALT, AST, AP), and renal profile (Serum creatinine, eGFR, Electrolytes – Na, K, Cl), Urine sample (20 ml) will also be collected to analyse ACR. All individuals with elevated lipid levels will be advised by investigator on lifestyle modification including diet and physical activity.

4.6.2 Follow up data collection

All participants will be requested to visit the clinic in one-month intervals for next three months and anthropometric measures, SBP, DBP and pulse will be recorded over a three months period post randomization. Any AEs since the last visit and all the medications being taken by the participants will be recorded. Unused medication (IMP) will be collected and the drug compliance will be determined. During visit 05, all the laboratory investigations carried out at the visit 1 will be repeated.

4.7 Study duration

The study will be for a period of 3 months and the visits and the evaluations will be done as follows: screening (visit 1) and randomization (visit 2), 1 month (visit 3), 2 months (visit 4) and 3 months (visit 5) (Table 1).

Table 1: Data collection

	**Study period**				
	**Screening Visit 1**	**Recruitment Visit 2**	**Visit 3**	**Visit 4**	**Visit 5**
**Timepoint**	*-7 days*	Day 0	Day 30(+5)	Day 60(+5)	Day 90(+5)
Enrollment					
Eligibility Screen	X				
informed Consent	X				
Randomization		X			
Intervention					
Cinnamon 1000 mg / Placebo		X	X	X	
Assessments					
Medical history	X				
Anthropometric measurements	X	X	X	X	X
SBP, DBP and pulse	X	X	X	X	X
FBS	X				X
Hba1c	X				X
Lipid Profile[1]	X				X
Liver Profile[2]	X				X
Renal Profile[3]	X				X
Pregnancy Test & Contraception Review[4]	X	X	X	X	
Dispense study medication		X	X	X	
Check drug compliance			X	X	X
Check AE, SAE			X	X	X
Complete concomitant medications		X	X	X	X

[1] Total cholesterol, HDL, LDL, triglycerides; [2] AST, ALT, AP; [3] Serum creatinine, ACR, eGFR, electrolytes-Na, K Cl

[4] Pregnancy test at screening and ongoing review of contraception adherence applies to all women of child-bearing potential. If there is concern of pregnancy, test will be repeated

4.8 Discontinuation criteria

A subject can be discontinued from the study due to one or more of following reasons.

- Subject’s demand to discontinue the study
- Serious adverse events (SAEs) or unusual changes in clinical test results

- Principal investigator’s decision to terminate the study (low rates of compliance, complications or inability to tolerate the study for various reasons)

4.9 End of study

End of Study is defined as when the last subject has completed 12 weeks of treatment.

4.10 Termination of the entire trial

Premature termination of the clinical trial will be considered if the principal investigator believes it is necessary to terminate the clinical trial for safety reasons, or when the clinical trial proves to be impracticable.

5. INVESTIGATIONAL MEDICINAL PRODUCT

The treatment drug is SaaraLife OptiHeart - Ceylon Cinnamon extract capsule. It is a capsule containing a standardized *C. zeylanicum* bark extract as the active ingredient.

5.1 Formulation

The raw material (cinnamon bark) used in the production had been authenticated by the Industrial Technology Institute, Sri Lanka (ITI; Report No: CTS1715632). Fresh powdered cinnamon feed with particle size distribution ranging from 250 μm to 1 mm is fed to the extractor. Extraction is carried out multiple times with aqueous + ethanol solvent to ensure the recovery of the maximum content of active compounds. Once water and ethanol solvent are evaporated mixture is passed through an ethanol recovery unit for recovery of ethanol. Then the concentrated output is passed through a vacuum dryer at specific temperatures to obtain a powder (with minimum of 15% proanthocyanidins (PAC A). Finally, 500mg of this powder is packed into size 0 capsules. The capsule preparation was carried out at a manufacturing site with GMP (Good Manufacturing Practices) certification.

The placebo capsule will contain pharmaceutical grade wheat flour (tasteless, odourless white colour fine powder). The placebo oral capsule will be identical in shape, size, weight and texture to the *C. zeylanicum* oral capsule. In order to mask the aroma, cinnamon quills would be put into packets containing both the placebo and cinnamon capsules.

5.2 Packaging and Labelling

Labels will include: (a) name of the sponsor; (b) pharmaceutical dosage, route of administration, quantity of dosage units (and name/identifier of the product and strength/potency); (c) the code number to identify the contents and packaging operation; (d) the trial subject identification number, where applicable; (e) directions for use; (f) “for clinical trial use only”; (i) the storage conditions;

5.3 Storage and Handling

Cinnamon capsules should be stored at controlled room temperature below 25°C (77°F). Storage conditions are specified on the label. Until dispensed to the subjects, all bottles of study drugs should be stored in a securely locked area, accessible only to authorized site personnel.

To ensure the stability and proper identification, study drugs should not be stored in a container other than the container in which they were supplied. Consideration should be given to handling, preparation, and disposal through measures that minimize drug contact with the body.

5.4 Accountability for Study Drug

In accordance with GCP, the clinical unit will account for all study medication. The clinical unit are responsible for study medication accountability, reconciliation, and record maintenance.

Drug accountability records will be maintained during the study as follows:

- Amount of study medication received from the Sponsor
- Amount distributed to each patient and returned by them
- Amount of unused drug returned to the Sponsor or destroyed at Sponsors request

In addition, in the event of necessary disposal of opened but wasted medication, the disposal should be documented appropriately (i.e. witnessed), in accordance with applicable local regulations, and GCP procedures.

Participants are required to return used and unused bottles to the clinical unit. Storage bags will be provided to each patient. For all unused study medication, the patient should adhere to the storage instructions until the study medication is returned to the clinical unit.

5.5 Investigational Medicinal Product Return or Disposal

Following accountability of study medication at the study site and on authorization of Coordinating Center, study medication will be returned to originator for destruction.

6. SAFETY ASSESSMENT

6.1 Definitions of Adverse Events, Adverse Reactions, and Serious Adverse Events

AE	Any untoward medical occurrence in a participant to whom a medicinal product has been administered, including occurrences which are not necessarily caused by or related to that product.
Adverse Reaction (AR)	An untoward and unintended response in a participant to an investigational medicinal product which is related to any dose administered to that participant.<br><br>The phrase "response to an investigational medicinal product" means that a causal relationship between a trial medication and an AE is at least a reasonable possibility, i.e. the relationship cannot be ruled out.<br><br>All cases judged by either the reporting medically qualified professional or the Sponsor as having a reasonable suspected causal relationship to the trial medication qualify as adverse reactions.
SAE	A SAE is any untoward medical occurrence that:<br>• results in death<br>• is life-threatening<br>• requires inpatient hospitalisation or prolongation of existing hospitalisation<br>• results in persistent or significant disability/incapacity<br>• consists of a congenital anomaly or birth defect*.<br><br>Other ‘important medical events’ may also be considered a SAE when, based upon appropriate medical judgement, the event may

	jeopardise the participant and may require medical or surgical intervention to prevent one of the outcomes listed above.
Serious Adverse Reaction (SAR)	An AR that is both serious and, in the opinion of the reporting Investigator, believed with reasonable probability to be due to one of the trial treatments, based on the information provided.
Suspected Unexpected Serious Adverse Reaction (SUSAR)	A serious adverse reaction, the nature and severity of which is not consistent with the Reference Safety Information for the medicinal product in question set out:<br>• in the case of a product with a marketing authorisation, in the approved summary of product characteristics (SmPC) for that product<br>• in the case of any other investigational medicinal product, in the approved investigator's brochure (IB) relating to the trial in question.

6.2 Assessment of Adverse Events and Serious Adverse Events

Assessment of Causality for Study Drugs and Procedures

The relationship of each AE to the trial medication must be determined by the site the principal investigator according to the following definitions:

Related: The AE follows a reasonable temporal sequence from trial medication administration. It cannot reasonably be attributed to any other cause.

Not Related: The AE is probably produced by the participant's clinical state or by other modes of therapy administered to the participant.

Assessment of Severity

Intensity/Severity of an AE will be graded as mild, moderate, or severe by the investigator based on her/his medical judgment and the following guidance:

Mild: asymptomatic or mild symptoms; clinical or diagnostic observations only; intervention not indicated.

Moderate: minimal, local or non-invasive intervention indicated; limiting age-appropriate instrumental activities of daily living (e.g. preparing meals, shopping for groceries or clothes, using the telephone)

Severe: medically significant but not immediately life-threatening; hospitalization or prolongation of hospitalization indicated; disabling; limiting self-care activities of daily living (e.g. bathing, dressing and undressing, feeding self).

6.3 Investigator Requirements and Instructions for Reporting Adverse Events and Serious Adverse Events

All SAEs must be reported on the SAE reporting form to the principal investigator within 24 hours of the Central Study Team becoming aware of the event. SAEs that are reported late must be accompanied by an explanation for this. The principal investigator will perform an initial check of the report and request any additional information from the Central Study Team.

The following information will be reported on the CRF: description, date of onset and end date, severity, assessment of relatedness to trial medication, other suspect drug or device and action taken. Follow-up information should be provided as necessary.

Site investigator/s will adhere to local ethical requirements of safety reporting. Such AE/SAE conditions will be followed up by the investigator until the issue is resolved.

7. DATA MANAGEMENT

Data collection: data collection will be carried out by trained research coordinators, using a standardized case record form, at screening (Visit '1'), randomization (Visit '2') and during each monthly follow up visit (Visit '3' and '4′) and EOS (Visit '5′). After filling out the CRF, data collection will be performed according to the standard operating procedures (SOPs). Storage: data will be entered into a local database by a minimum number of dedicated staff and saved in a dedicated computer with password protection.

8. STATISTICAL CONSIDERATION

8.1 Sample size

The sample size was calculated assuming that the IMP would reduce total cholesterol by 10 mg/dL using below formula [31]. Assuming a standard deviation of 40 for total cholesterol and a correlation of 0.9 between the baseline and three months measurement a sample size of 64 per group would give 90% power at a significance level of 0.05. Expecting a 15% dropout rate the final sample size required will be 150 (75 per arm).

$$N \approx 4\sigma^2(1-\rho^2)(z_{1-\frac{\alpha}{2}} + z_{1-\beta})^2/\Delta^2$$

8.2 Statistical Analysis

Reduction in total cholesterol level at three months (from the baseline value) will be compared between the two groups – cinnamon vs placebo – using ANCOVA. Intention to treat analysis will be adhered to in analysing the outcome data. Data analysis will be done using version 4.0 of the R statistical software.

9. INFORMED CONSENT

The participant must personally sign and date the latest approved version of the Informed Consent form before any trial specific procedures are performed.

Written and verbal versions of the Participant Information and Informed Consent will be presented to the participants detailing no less than: the exact nature of the trial; what it will involve for the participant; the implications and constraints of the protocol; the known side effects and any risks involved in taking part. It will be clearly stated that the participant is free to withdraw from the trial at any time for any reason without prejudice to future care, without affecting their legal rights and with no obligation to give the reason for withdrawal.

The participant will be allowed as much time as wished to consider the information, and the opportunity to question the Investigator. Written Informed Consent will then be obtained by means of participant dated signature and dated signature of the person who presented and obtained the Informed Consent. The person who obtained the consent must be suitably qualified and experienced and have been authorised to do so by an Investigator. A copy of the signed Informed Consent will be given to the participant. The original signed form will be retained at the trial site.

10. RESPONSIBILITIES

Responsibilities of investigator/s

The investigator will ensure that this study is conducted in accordance with the principles of the Declaration of Helsinki, International Council for Harmonisation (ICH) guidelines, or with the laws and regulations of the country in which the research is conducted, whichever affords the greater protection to the study subject.

The investigator will submit this protocol, ICF, and any accompanying material to be provided to the subject (such as advertisements, subject information sheets, or descriptions of the study used to obtain informed consent) to an ERC. The investigator will not begin any study subject activities until approval from the ERC has been documented and provided as a letter to the investigator.

The investigator is responsible for obtaining written informed consent from each individual participating in this study after adequate explanation of the aims, methods, objectives, and potential hazards of the study and before undertaking any study-related procedures.

The investigator must assure that subjects’ anonymity will be strictly maintained and that their

identities are protected from unauthorized parties. Only subject initials, date of birth, another unique identifier (as allowed by local law) and an identification code will be recorded on any form or biological sample submitted to the Sponsor, ERC, or laboratory.
The investigator is responsible for ensuring the study is conducted in accordance with the procedures and evaluations described in this protocol.

11. DISSEMINATION OF RESULTS

Primary and secondary outcomes of the study will be disseminated via research publications.

12. CONFIDENTIALITY

All documents and data relating to this trial are strictly confidential. Documents given to the investigators and trial site by the Coordinating Center should not be disclosed to other parties without the written approval of the sponsor. The investigator/delegates should maintain the confidentiality of the identification of all trial participants and assure the security and confidentiality of trial data and documents.
Personal identifiers will be removed, and the anonymity of participants will be secured through both research unit codes and pseudonyms.

13. ETHICAL CONSIDERATION

This research project will be conducted with full compliance of research ethics norms, the Declaration of Helsinki and the GCP guidelines. Ethical clearance will be obtained from an Ethics Review Committee affiliated to the Faculty of Medicine, University of Kelaniya, Sri Lanka. The trial will also be registered at the Sri Lanka Clinical Trials Registry.
Any change in trial protocol will be notified to the relevant regulatory authorities and trial participants, with re-consent being taken from participants, if required.

14. REFERENCES

1. Ibrahim M.A., Asuka E. and Jialal I. (2020). Hypercholesterolemia. https://www.ncbi.nlm.nih.gov/books/NBK459188/ (Accessed on 15.09.2020)
2. Ceylon Med J, 2016. 61(1): p. 11-7.Sando KR, Knight M. Nonstatin therapies for management of dyslipidemia: A review. Clin Ther. 2015;37:2153–79.
3. Robinson JG. Management of familial hypercholesterolemia: A review of the recommendations from the national lipid association expert panel on familial hypercholesterolemia. J Manag Care Pharm. 2013;19:139–49.
4. Trentman, T. L., Avey, S. G., & Ramakrishna, H. (2016). Current and emerging treatments for hypercholesterolemia: A focus on statins and proprotein convertase subtilisin/kexin Type 9 inhibitors for perioperative clinicians. *Journal of anaesthesiology, clinical pharmacology*, *32*(4), 440.
5. Paththinige C, Sirisena N, Dissanayake V. Genetic determinants of inherited susceptibility to hypercholesterolemia – a comprehensive literature review. Lipids in Health and Disease. 2017;16(1).
6. Bilen O, Kamal A, Virani S. Lipoprotein abnormalities in South Asians and its association with cardiovascular disease: Current state and future directions. World Journal of Cardiology. 2016;8(3):247.
7. Katulanda P, Dissanayake H, De Silva S, Katulanda G, Liyanage I, Constantine G et al. Prevalence, patterns, and associations of dyslipidemia among Sri Lankan adults—Sri Lanka Diabetes and Cardiovascular Study in 2005–2006. Journal of Clinical Lipidology. 2018;12(2):447-454.
8. Ranasinghe P, Jayawardana R, Galappaththy P, Constantine GR, de Vas GN, Katulanda P. Efficacy and safety of "true" cinnamon (*Cinnamomum zeylanicum*) as a pharmaceutical agent in diabetes: a systematic review and meta-analysis. Diabet Med. 2012;29(12):1480–92.
9. Lungarini S, Aureli F, Coni E. Coumarin and cinnamaldehyde in cinnamon marketed in Italy: a natural chemical hazard? Food Addit Contam Part A Chem Anal Control Expo Risk Assess. 2008;25(11):1297–305.

10. Ghosh P, Markin RS, Sorrell MF. Coumarin-induced hepatic necrosis. Am J Gastroenterol. 1997;92(2):348–9.
11. Shen Q, Chen F, Luo J. Comparison studies on chemical constituents of essential oil from ramulus cinnamomi and cortex cinnamomi by GC-MS. Zhong Yao Cai. 2002;25:257–258.
12. R. Hamidpour, M. Hamidpour, S. Hamidpour, and M. Shahlari, "Cinnamon from the selection of traditional applications to its novel effects on the inhibition of angiogenesis in cancer cells and prevention of Alzheimer's disease, and a series of functions such as antioxidant, anticholesterol, antidiabetes,antibacterial, antifungal, nematicidal, acaracidal, and repellent activities," Journal of Traditional and Complementary Medicine, vol. 5, no. 2, pp. 66–70, 2015
13. Singh G, Maurya S, de Lampasona M, Catalan C. A comparison of chemical, antioxidant and antimicrobial studies of cinnamon leaf and bark volatile oils, oleoresins and their constituents. Food and Chemical Toxicology. 2007;45(9):1650-1661.
14. Rao P, Gan S. Cinnamon: A Multifaceted Medicinal Plant. Evidence-Based Complementary and Alternative Medicine. 2014;2014:1-12.
15. Volgman A, Palaniappan L, Aggarwal N, Gupta M, Khandelwal A, Krishnan A et al. Atherosclerotic Cardiovascular Disease in South Asians in the United States: Epidemiology, Risk Factors, and Treatments: A Scientific Statement From the American Heart Association. Circulation. 2018;138(1).
16. Gupta M, Brister S, Verma S. Is South Asian ethnicity an independent cardiovascular risk factor?. Canadian Journal of Cardiology. 2006;22(3):193-197.
17. Katulanda, P., Dissanayake, H. A., De Silva, S. N., Katulanda, G. W., Liyanage, I. K., Constantine, G. R., ... & Matthews, D. R. (2018). Prevalence, patterns, and associations of dyslipidemia among Sri Lankan adults—Sri Lanka Diabetes and Cardiovascular Study in 2005–2006. *Journal of Clinical Lipidology*, *12*(2), 447-454.
18. Non communicable diseases in Sri Lanka. WHO Country Profiles: Sri Lanka 2014 [cited; Available from: http://www.who.int/nmh/countries/lka_en.pdf
19. Ranawaka, U.K., et al., Risk estimates of cardiovascular diseases in a Sri Lankan community.

20. Hassan, S. A., Barthwal, R., Nair, M. S., & Haque, S. S. (2012). Aqueous bark extract of Cinnamomum zeylanicum: a potential therapeutic agent for streptozotocin-induced type 1 diabetes mellitus (T1DM) rats. *Tropical Journal of Pharmaceutical Research, 11*(3), 429-435.
21. Muhammad, F., Aslam, B., Ahmad, M., & Shahzadi, A. (2012). Lipid lowering effect of Cinnamomum zeylanicum in hyperlipidaemic albino rabbits. *Pak. J. Pharm. Sci*, *25*(1), 141-147.
22. Trentman, T. L., Avey, S. G., & Ramakrishna, H. (2016). Current and emerging treatments for hypercholesterolemia: A focus on statins and proprotein convertase subtilisin/kexin Type 9 inhibitors for perioperative clinicians. Journal of anaesthesiology, clinical pharmacology, 32(4), 440.
23. Abdelgadir, A. A., Hassan, H. M., Eltaher, A. M., Khnsaa Mohammed, G. A., Lamya Mohammed, A. A., & Hago, T. B. (2020). Hypolipidemic Effect of Cinnamon (Cinnamomum zeylanicum) Bark Ethanolic Extract on Triton X-100 induced Hyperlipidemia in Albino Rats. *Med Aromat Plants (Los Angeles)*, *9*(351), 2167-0412.
24. Ranasinghe, P., Jayawardena, R., Pigera, S., Wathurapatha, W. S., Weeratunga, H. D., Premakumara, G. S., ... & Galappaththy, P. (2017). Evaluation of pharmacodynamic properties and safety of Cinnamomum zeylanicum (Ceylon cinnamon) in healthy adults: a phase I clinical trial. BMC complementary and alternative medicine, 17(1), 550.
25. Gupta Jain S, Puri S, Misra A, Gulati S, Mani K. Effect of oral cinnamon intervention on metabolic profile and body composition of Asian Indians with metabolic syndrome: a randomized double -blind control trial. Lipids in Health and Disease. 2017;16(1).
26. Khan A, Safdar M, Ali Khan M, Khattak K, Anderson R. Cinnamon Improves Glucose and Lipids of People With Type 2 Diabetes. Diabetes Care. 2003;26(12):3215-3218.
27. Eidi, A., Mortazavi, P., Bazargan, M., & Zaringhalam, J. (2012). Hepatoprotective activity of cinnamon ethanolic extract against CCI4-induced liver injury in rats. Excli Journal, 11, 495.
28. Askari F, Rashidkhani B, Hekmatdoost A. Cinnamon may have therapeutic benefits on lipid profile, liver enzymes, insulin resistance, and high-sensitivity C-reactive protein in nonalcoholic fatty liver disease patients. Nutrition Research. 2014;34(2):143-148.

29. Grundy S, Stone N, Bailey A, Beam C, Birtcher K, Blumenthal R et al. 2018 AHA/ACC/AACVPR/AAPA/ABC/ACPM/ADA/AGS/APhA/ASPC/NLA/PCNA Guideline on the Management of Blood Cholesterol. Journal of the American College of Cardiology. 2019;73(24):e285-e350.
30. CRAN - Package blockrand [Internet]. Cran.r-project.org. 2020 [cited 20 September 2020]. Available from: https://CRAN.R-project.org/package=blockrand
31. Morgan, T. M., & Case, L. D. (2013). Conservative sample size determination for repeated measures analysis of covariance. Annals of biometrics & biostatistics, 1(1), 1002.
