# Supplementary material for: Effects of Cinnamomum zeylanicum (Ceylon cinnamon) extract on lipid profile, glucose levels and its safety in adults: A randomized, double-blind, controlled trial
Source: PLoS One. 2025 Jan 24;20(1):e0317904. doi: 10.1371/journal.pone.0317904 (PMC11759401; doi:10.1371/journal.pone.0317904)
Supplement: Attachment — Submitted filename: PONE-D-24-00185_Responses to reviewer comments.pdf [file pone.0317904.s004.pdf]

AUTHOR RESPONSES TO EDITORS AND REVIEWERS

Please note that page and paragraph numbers are in reference to the location of changes in the clean, untracked version of the manuscript.

Editorial Comments:

1. **Please ensure that your manuscript meets PLOS ONE's style requirements, including those for file naming. The PLOS ONE style templates can be found at:**

   https://journals.plos.org/plosone/s/file?id=wjVg/PLOSOne_formatting_sample_main_body.pdf

   https://journals.plos.org/plosone/s/file?id=ba62/PLOSOne_formatting_sample_title_authors_affiliations.pdf

   Response:
   We have ensured that the manuscript now adheres to PLOS ONE's style requirements. The list of figures and tables has been included at the end of the document.

2. **Please note that funding information should not appear in any section or other areas of your manuscript. We will only publish funding information present in the Funding Statement section of the online submission form. Please remove any funding-related text from the manuscript.**

   Response:
   The funding information has been removed from the manuscript, and we have ensured that it only appears in the Funding Statement section of the online submission form.

3. **Thank you for stating the following financial disclosure: "Financial support for this research was provided by SDS Spices Pvt Ltd." Please state what role the funders took in the study. If the funders had no role, please state: "The funders had no role in study design, data collection and analysis, decision to publish, or preparation of the manuscript." If this statement is not correct you must amend it as needed. Please include this amended Role of Funder statement in your cover letter; we will change the online submission form on your behalf.**

   Response:
   We confirm that the funders had no role in the study design, data collection and analysis, decision to publish, or preparation of the manuscript.

4. **Thank you for stating the following in the Competing Interests section: "I have read the journal's policy and the authors of this manuscript have the following competing interests: Financial support for this research was provided by SDS Spices Pvt Ltd. This funding covered the costs associated with staff at research sites, including the reimbursement of authors Dimuthu Muthukuda and Arunasalam Pathmeswaran for the time devoted to this study. The authors declare that this funding did not influence the study design, data collection, analysis, interpretation, or the decision to publish these findings." Please confirm that this does not alter your adherence to all PLOS ONE policies on sharing data and**

materials, by including the following statement: "This does not alter our adherence to PLOS ONE policies on sharing data and materials." (as detailed online in our guide for authors http://journals.plos.org/plosone/s/competing-interests). If there are restrictions on sharing of data and/or materials, please state these. Please note that we cannot proceed with consideration of your article until this information has been declared. Please include your updated Competing Interests statement in your cover letter; we will change the online submission form on your behalf.

Response:
We confirm that the statement does not alter our adherence to PLOS ONE policies on sharing data and materials. This is included in the cover letter.

5. **In the online submission form, you indicated that "The data that support the findings of this study are available from the corresponding author, S.A, upon reasonable request." All PLOS journals now require all data underlying the findings described in their manuscript to be freely available to other researchers, either 1. In a public repository, 2. Within the manuscript itself, or 3. Uploaded as supplementary information. This policy applies to all data except where public deposition would breach compliance with the protocol approved by your research ethics board. If your data cannot be made publicly available for ethical or legal reasons (e.g., public availability would compromise patient privacy), please explain your reasons on resubmission and your exemption request will be escalated for approval.**

   Response:
   We have now updated the manuscript to include the data as supplementary material.

6. **Your ethics statement should only appear in the Methods section of your manuscript. If your ethics statement is written in any section besides the Methods, please delete it from any other section.**

   Response:
   The ethics statement has been updated and is now only included in the Methods section of the manuscript (lines 81 – 83).

7. **Please ensure that you refer to Figure 2 in your text as, if accepted, production will need this reference to link the reader to the figure.**

   Response:
   We have updated the manuscript to refer to Figure 2 in the appropriate section (line 306).

8. **Please include a separate caption for each figure in your manuscript.**

   Response:
   Separate captions have been included for each figure in the manuscript.

Reviewer 1 Comments:

1. **For the sample size calculation, whether a formula or sample size calculator/software was used to derive the sample size is to be stated.**

   Response: We have updated the manuscript to include details of the formula used for the sample size calculation (line 168).

2. **The information on the effect size, how the correlation coefficient of 0.9 between baseline and six-month measurements was incorporated into the sample size calculation, and the sample size before the attrition rate 15% is to be stated.**

   Response: We have updated the manuscript to include detailed information regarding the effect size, the incorporation of the correlation coefficient of 0.9 between baseline and six-month measurements into the sample size calculation, and the sample size before accounting for the 15% attrition rate. These details are now provided in the revised Methods section (lines 159 – 175).

3. **Line 173-174, although complete case analysis is easy to implement, it can contribute to biased results if the missing data are not missing completely at random (MCAR). Perhaps a statement on handling missing data could be added by exploring the pattern of missing data and the possible imputation method, model-based approaches etc if required.**

   Response: Little's MCAR test was performed to assess the missing data pattern. It indicated that the data were missing completely at random (MCAR), allowing us to proceed with a complete-case analysis for the outcome data. The manuscript has been updated to reflect this information (lines 210 – 212).

4. **Line 172-174, the ANCOVA analysis is unclear. More details are to be provided. The dependent variable, independent variable(s), and covariate variable are to be stated. Whether the dependent variable refers to LDL-C at 3 months or changes from baseline to 3 months is to be clearly stated. From Table 2 presentation, it indicates the dependent variable LDL-C is at 3 months.**

   Response: We have provided additional details on the ANCOVA analysis, including the dependent variable (eg: LDL-C at 3 months), independent variables, and covariates (lines 212 – 214).

5. **A statement on the fulfillment of ANCOVA assumptions is to be provided.**

   Response: We have added a statement regarding the fulfillment of ANCOVA assumptions in the manuscript (lines 214 – 217).

6. **Line 176-177, the treatment and potential subgroups are to be mentioned and the name of statistical test that will be employed is to be mentioned.**

   Response: We have updated the manuscript to include information about the treatment, potential subgroups, and the statistical test employed (lines 220 – 225).

7. **Line 178, the actual citation for the statistical software R is to be provided.**

   Response: We have included the citation for the statistical software R in the manuscript (lines 226).

8. **Table 1 Female, typo 6.07%.**

   Response: The typo has been corrected, and the value is now updated to 67.0% (Table 1).

9. **Table 1, for the glycemic status variable, the subcategories are not to be directly below the same alignment with the glycemic status available. Since symbol % has been indicated in the first column of those variables, all individual symbol % could be omitted.**

   Response: The alignment and formatting of the glycemic status variable in Table 1 have been corrected, and the redundant % symbols have been removed.

10. **Table 2, the table requires cosmetic changes and to follow the journal requirement. Mean, SD, 95% CI, statistical test is to be denoted in the table footnote. For Hb available, AST, eGFR, ACR, BMI for p >0.9, the actual p-value is to be provided. The decimal point for the p-value is to be standardized in the table and text. One decimal point is to be avoided. This applies to others where applicable. The word mean fall is to be avoided and replaced with another word.**

    Response: We have made the necessary cosmetic changes to Table 2, ensuring it aligns with the journal's requirements. The table footnotes now denote Mean, SD, 95% CI, and statistical tests. Actual p-values for p >0.9 have been provided, and the decimal points have been standardized throughout. The term "mean fall" has been replaced with “mean reduction”.

11. **Line 212, P=0.036 to be replaced as p=0.036.**

    Response: This has been corrected in the manuscript (line 263).

12. **Line 215-220, the adjusted findings are to be “discussed” as well apart from the unadjusted findings.**

    Response: We have updated the manuscript to discuss the adjusted findings alongside the unadjusted findings (lines 271 – 276).

13. **Effect size indices could be presented.**

    Response: Cohen’s d values have been calculated and added to Table 2 in the manuscript.

14. **Table 3, how the independent variable was coded for the multivariate linear regression is to be stated. For the adjusted analysis, model fit is to stated.**

Response: We have updated the methods section (line 208 – 209) to include coding for the independent variable and provided information on model fit for the adjusted analysis in Table 3.

15. **Table 3, Beta is to be labeled as standardized coefficients and denoted in the table footnote.**

    Response: We have labeled Beta as standardized coefficients and updated the table footnote accordingly.

16. **Table 3 anthropometrics and vitals (for p >0.9), the actual p-value is to be presented. Similarly, the decimal point for the p-value, beta coefficient and 95%CI is to be standardized. One decimal point for the p-value is to be avoided. This applies to others where applicable.**

    Response: The formatting of p-values, beta coefficients, and 95% CI in Table 3 has been standardized.

17. **Line 221, multivariate linear regression is to be stated in the statistical analyses section. The use of multivariate linear regression is to be clearly stated, e.g. whether the focus is on estimating treatment effects at follow-up etc and whether adjusting for baseline values is required.**

    Response: We have clarified in the manuscript that multiple linear regression, rather than multivariate regression, was employed in the analysis. The focus was on estimating the treatment effects on LDL-C levels and other clinical parameters, specifically concentrating on the absolute change from baseline to 3 months. Consequently, changes in these parameters were not adjusted for baseline values in the analysis (line 285).

18. **Line 221-222, the word 'at 3 months' is to be stated.**

    Response: The phrase "at 3 months" has been added to the Table 3 caption (lines 285 – 287).

19. **Line 239, (-63), (95% CI to be presented as (-63, 95%C….).**

    Response: The 95% CI has been presented as (-63, 95% CI…) in the text (lines 310 – 311).

20. **Line 246, the timepoint baseline -3 months is to be stated.**

    Response: We have stated the timepoint as baseline to 3 months (lines 317 - 319).

21. **Line 254, the timepoint or changes are to be stated.**

    Response: The caption for Table 6 has been updated to capture that changes in fasting blood sugar levels from baseline to 3 months were considered for linear regression (lines 324 - 326).

22. **Table 5 is to be cited in the text.**

    Response: Table 5 is now cited in the text (line 306).

23. **Table 6, the decimal point for the p-value is to be standardized. One decimal point is to be avoided**.

    Response: The decimal point for the p-value in Table 6 has been standardized.

24. **References did not adhere to the format specified by the journal.**

Response: The references have been reformatted to adhere to the journal's specifications.

Reviewer 2 Comments

1. **Your Keywords should written based on MeSH term.**

   Response: The keywords have been updated to align with MeSH terms as follows: Cinnamomum zeylanicum; Randomized Controlled Trial; Phytotherapy; Low-Density Lipoprotein Cholesterol; Diabetes Mellitus.

2. **In the introduction, you should first discuss the role of complementary therapies (natural compounds) products in human health and chronic diseases prevention and treatment, also its role in improving glycemic indices and lipid profile levels in adults, then discuss the probable role of cinnamon in glycemic and lipid profile indices. You can also use the following references in your article for strengthen the introduction and discussion sections. These references are helpful.**

   1) **Ranasinghe, P., Galappaththy, P., Constantine, G. R., et al. (2017). Cinnamomum zeylanicum (Ceylon cinnamon) as a potential pharmaceutical agent for type-2 diabetes mellitus: study protocol for a randomized controlled trial. Trials, 18, 1-8.**
   2) **Gheflati et al. The effects of cinnamon supplementation on adipokines and appetite-regulating hormones: A systematic review of randomized clinical trials.**
   3) **The effect of conjugated linoleic acids on inflammation, oxidative stress, body composition and physical performance: a comprehensive review of putative molecular mechanisms (Nutrition & Metabolism).**
   4) **Does propolis have any effect on rheumatoid arthritis? A review study (Food Science & Nutrition).**
   5) **Nattagh-Eshtivani et al. The role of Pycnogenol in the control of inflammation and oxidative stress in chronic diseases: Molecular aspects.**
   6) **Effects of Momordica charantia L on blood pressure: a systematic review and meta-analysis of randomized clinical trials (International Journal of Food Properties).**
   7) **Bahari et al. The effect of Royal jelly on liver enzymes and glycemic indices: A systematic review and meta-analysis of randomized clinical trials.**
   8) **Barghchi et al. The effects of Chlorella vulgaris on cardiovascular risk factors: A comprehensive review on putative molecular mechanisms.**

   Response: We have expanded the introduction to discuss the role of complementary therapies and natural compounds in human health and chronic disease prevention (lines 36 – 48), as well as the role of cinnamon in improving glycemic indices and lipid profile levels (lines 385 – 404). We

have incorporated some of the suggested references to strengthen the introduction and discussion sections.

3. **Please more explain about randomization and blinding.**

   Response: Additional details on randomization and blinding have been included in sections 2.3 and 2.4 (lines 108 – 130).

4. **Please mention your sample size calculation formula and related study.**

   Response: The sample size calculation formula and relevant study have been added to the text (lines 159 – 175).

5. **Have you considered medication intake for this study? And did you adjust their effects?**

   Response: Participants currently on statins or other lipid-lowering drugs were excluded from the study to control for potential confounding effects of these medications on lipid levels (line 99). The study design did not include adjustments for medication effects beyond this exclusion criterion. Therefore, the potential impact of other medication types or dosage variations on the study outcomes was not specifically addressed in the analysis. Future studies could consider incorporating a more detailed assessment of medication use and its potential effects on clinical outcomes.

6. **Dietary intake (carbohydrates, protein, fat) as well as some micronutrients such as omega-3 fatty acids can affect blood sugar and lipid profile. Why did you not report food intake? (Please report it in table).**

   Response: Dietary intake data, including carbohydrates, protein, fat, and micronutrients such as omega-3 fatty acids, was not recorded as part of this study. The primary focus was to evaluate the specific impact of *Cinnamomum zeylanicum* on LDL-C levels. Incorporating detailed dietary assessments would have required additional resources and complexity beyond the scope of this study.

   We acknowledge that dietary factors can influence blood sugar and lipid profiles. However, the study design and analysis focused on assessing the direct effects of the intervention, and potential confounding factors related to diet were not addressed in this phase of the research. Future studies with a broader scope could include dietary assessments to explore their interactions with the intervention more thoroughly.

7. **The discussion should be expanded to include additional references and a more detailed explanation of the probable mechanisms of action of the compound. Ensure that any grammatical errors are corrected.**

   Response: The discussion has been expanded with additional references and a more detailed explanation of the probable mechanisms of action of the compound. We have also improved the grammatical errors.
