# Supplementary material for: Effects of Cinnamomum zeylanicum (Ceylon cinnamon) extract on lipid profile, glucose levels and its safety in adults: A randomized, double-blind, controlled trial
Source: PLoS One. 2025 Jan 24;20(1):e0317904. doi: 10.1371/journal.pone.0317904 (PMC11759401; doi:10.1371/journal.pone.0317904)
Supplement: Attachment — Submitted filename: PONE-D-24-00185R1_Responses to reviewer comments.pdf [file pone.0317904.s005.pdf]

AUTHOR RESPONSES TO EDITORS AND REVIEWERS

Please note that page and paragraph numbers are in reference to the location of changes in the clean, untracked version of the manuscript.

Editorial Comments:

1. **Please review your reference list to ensure that it is complete and correct. If you have cited papers that have been retracted, please include the rationale for doing so in the manuscript text, or remove these references and replace them with relevant current references. Any changes to the reference list should be mentioned in the rebuttal letter that accompanies your revised manuscript. If you need to cite a retracted article, indicate the article's retracted status in the References list and also include a citation and full reference for the retraction notice.**

   Response:

   We have thoroughly reviewed the reference list to ensure its completeness and accuracy. We confirm that no cited papers have been retracted, and therefore, no changes to the reference list were necessary.

Reviewer 1 Comments:

1. **For Table 2, the 95% confidence interval for Cohen D is to be denoted in the table footnote.**

   Response: The 95% confidence interval for Cohen's d has been added to the table footnote in Table 2. The update is reflected on line 254 of the revised manuscript.

Reviewer 3 Comments

1. **English Language and formatting consistency:**

- **The English language used throughout the manuscript is generally clear and appropriate for a scientific publication. However, there are a few instances where the phrasing could be improved for clarity, such as the sentences in lines 378-384. Please revise carefully the whole manuscript.**
- **The manuscript would benefit from a thorough review of grammar, punctuation, and capitalization to ensure consistency throughout the document. For example, the term "Cinnamomum zeylanicum" should be capitalized consistently, but cinnamon shouldn't (see, for example, line 46).**

Response:
We have carefully revised the manuscript to improve clarity and ensure consistency in grammar, punctuation, and capitalization throughout. Specifically:

- Sentences in lines 377–384 have been rephrased for better clarity.
- The term *Cinnamomum zeylanicum* is now consistently capitalized, while "cinnamon" remains lowercase, as appropriate.

2. **Abstract:**

- **In the objective section, you mention evaluating LDL-c levels, but you also measured other parameters, such as fasting blood sugar (FBS), that you mention on the results. It would be helpful to clarify the outcome of interest in the objective.**

  Response:
  The objective section has been updated to clarify the primary and secondary outcomes of the study. The revised text, on lines 6-11 now, specifies that the study primarily aimed to evaluate the effect of a standardized *C. zeylanicum* extract on serum LDL-C levels and secondarily on other lipid parameters, glucose levels, anthropometric measures, blood pressure, and safety outcomes.

- **Could you please provide the justification for choosing the 1000 mg/day dose of Cinnamomum zeylanicum extract? This information should also be included in the methods section (lines 149-150).**

  Response:

  This dose was chosen based on prior preclinical and clinical studies suggesting potential efficacy and safety. The justification for selecting the 1000 mg/day dose of *C. zeylanicum* extract has now been included in the methods section of the abstract (lines 15-16) and main text (lines 149-150).

- **The statement about a "significant interaction effect" (lines 22-23) could use some additional clarification.**

  Response:
  The statement about a "significant interaction effect" has been clarified for better understanding. The updated text on lines 26-30 explains that individuals with T2DM who received *C. zeylanicum* extract experienced a notable reduction in FBS levels.

- **The two concluding sentences in the abstract should not be separated by a period.**

  Response:

  The two concluding sentences in the abstract have been revised to ensure they are not separated by a period. The updated text is on lines 32-34.

3. **Introduction:**

- **The reference for the sentence in line 42 is missing. Could you please provide the appropriate citation?**

  Response:
  The reference for the sentence has been added, and the citation is now included on line 47 of the revised manuscript.

- **It would be interesting to know the potential advantages of using *Cinnamomum zeylanicum*, which has a lower coumarin content, and the rationale for conducting this study.**

  Response:

  We have updated the manuscript (lines 66-67) to include the potential advantages of using *C. zeylanicum* due to its lower coumarin content. The rationale for conducting this study (lines 74-82) has been re-worded for better clarity.

4. **Materials and methods:**

- **Please double-check the amount of PAC A per 100g of the cinnamon extract reported in line 142 (15298 mg of PAC A per 100g) and revise if necessary.**

  Response:
  We have double-checked the reported amount of PAC A per 100g of the cinnamon extract (15,298 mg of PAC A per 100g) and confirm that these details are correct.

- **For clarity, it would be helpful to define the abbreviations used in the manuscript, such as IMP (line 158), HbA1c (line 186), SBP and DBP (line 188), AST, ALT, and eGFR (line 190). Please revise all the abbreviations throughout the text.**

  Response:
  The abbreviations used in the manuscript, including IMP (line 142), HbA1c (line 198), SBP and DBP (line 200), AST, ALT, and eGFR (line 202-203), have been defined for clarity. We have updated the entire document to ensure consistency in abbreviation usage.

5. **Results:**

- **Lines 238-239: It is unclear what the "normal levels at baseline" were for the outcomes presented. Please specify the actual baseline values and what is considered the normal/healthy range for these measures.**

  Response:
  The actual baseline values are now included on lines 249-251. The normal/healthy ranges for these measures have not been included in the current manuscript as they are not the focus of the analysis.

- **The presentation of Tables 2 and 3 should be improved. The number of participants per group should be clearly stated in the tables rather than using a footnote. Additionally, the formatting of the data (e.g. "Mean (SD); n (%)") should be consistent across all parameters.**

  Response:
  The presentation of Tables 2 and 3 has been enhanced for better clarity. The baseline clinical parameters of the participants in each group have been moved from Table 2 to Table 1. The number

of participants per group is now clearly stated within the tables, and the formatting of the data (e.g., "Mean (SD); n (%)") has been made consistent across all parameters.

- **Lines 267-269: please provide the actual numerical values that support the conclusions drawn from the results, rather than just stating the observations qualitatively.**

  Response:
  The actual numerical values supporting the conclusions drawn from the results in lines 267-269 have been provided, and the updated text can be found on lines 293-294.

- **The quality of both figures should be improved to ensure the data is clearly visible and interpretable.**

  Response:
  The quality of both figures has been enhanced to ensure the data is clearly visible and interpretable.

6. **Discussion:**

- **Consistency of period: Throughout the manuscript, you refer to the trial duration as both 3 months and 12 weeks. While these are equivalent, it would be best to use a single, consistent period to avoid any potential confusion.**

  Response:
  To ensure consistency, the trial duration has been updated to "12 weeks" throughout the entire manuscript, replacing all instances of "3 months."

- **Strengths and limitations: I would recommend mentioning the key strengths and limitations of your study together at the end of the discussion section. This will help provide the reader with a clear overview of the merits and potential shortcomings of your work.**

  Response:

  The key strengths and limitations of the study have been added together at the end of the discussion section, providing a clear overview of the merits and potential shortcomings of the work. The relevant text can be found on lines 431-439.

- **Rationale for dose selection: In the discussion, you compare your results to studies using different dosages of cinnamon extract. It would be helpful to explicitly explain in the introduction or methods why you selected the 1000 mg/day dose for your trial.**

  Response:

  The justification for selecting the 1000 mg/day dose of *C. zeylanicum* extract has been included in the methods section (lines 149-150). This dose was chosen based on prior preclinical and clinical studies suggesting potential efficacy and safety.

- **Mechanism of action: Your discussion of the putative mechanism of action for the cinnamon extract effects is interesting. If possible, can you provide any information on the specific molecules present in the extract that may be responsible for these effects?**

  Response:
  We have expanded the discussion to include more detailed information about proanthocyanidins (PACs), a compound found in *C. zeylanicum* extract. PACs are emerging as key components in modulating glucose homeostasis, and their potential role in diabetes management has been highlighted. The expanded discussion can be found in lines 394-403 of the manuscript. We hope this provides a clearer understanding of the molecules that may contribute to the observed effects of C. zeylanicum extract.

- **Liver and kidney profile details: When mentioning the liver and kidney profile findings, it would be useful to specify which parameters from Table 2 are related to these organ functions, as some readers may not be familiar with interpreting these results.**

  Response:
  When discussing the liver and kidney profile findings, we have clarified which parameters from Table 2 are related to these organ functions. The updated text is on lines 404-406.

- **Missing data: Please clarify what you mean by "missing data" when discussing the study limitations.**

  Response:

  The clarification regarding "missing data" in the study limitations has been provided, and the updated text can be found on lines 435-437.
